# 

**Published:** 1903-07-25

**Authors:** 


### NOTICE TO CORRESPONDENTS.

All MSS., Letters, Books for Review, and other matters intended for the Editor should be addressed The Editor, "The Hospital," The Hospital Buildings, 28 & 29 Southampton Street, Strand, W.C.

The Editor cannot undertake to return rejected MSS., even when accompanied by stamped directed envelope.

**Notice to Advertisers and Subscribers.**

All Advertisements, Orders for Copies of The Hospital, and Business Communications should be addressed to **THE MANAGER, (Not to the Editor),** The Hospital, 28 & 29 Southampton Street, Strand, London, W.C.

The Cost of Subscription, post free, is as follows.—Per week, 3½d.; per quarter, 3s. 9d.; per half-year, 7s. 6d.; per annum, 15s. Foreign and Colonial:—Per annum, 19s. 6d., payable, in all cases, in advance.

**NOTICE.—Vol. XXXIII. of "The Hospital" (October 1902 to March 1903) in handsome cloth binding, will shortly be on sale at the office of this Journal, price 7s. 6d. Cases for binding the Numbers contained in this Volume 1s. 6d. Index to Vol. XXXIII., 2½d., post free.**

**The monthly part for August will be ready on August 1. Price 1s., by post 1s. 3d.**

### BOOKS RECEIVED.

T. FISHER UNWIN.

"A Woman's Wanderings during the Anglo-Boer War." By Mrs. General De la Rey.

J. WRIGHT AND CO., BRISTOL.

"Buxton, its Waters, Baths, etc." By Armstrong and Harburn.

THE UNIVERSITY TUTORIAL PRESS.

"Matriculation Directory No. XXXIV. June 1903."

JOHN WRIGHT AND CO.

"Lectures on Massage and Electricity." By T. Stretch Dowse, M.D.

EBLANA PRESS.

"Domestic Economy Reader." By Charlotte O'Connor Eccles.

MACMILLAN AND CO.

"Life and Labour of the People in London." By Charles Booth.

### Scraps and Gleanings.

Over 15 governments and over 100 municipalities are represented on the International Fire Prevention Congress, the members of which number some 500.

It is stated that the Vienna trade societies have resolved to arrange for an exhibition at Earl's Court in 1905 under the title "Austria in London."

The Hove Library Committee have accepted Mr. Carnegie's offer of £10,000 for a free library building for the town. Mr. Carnegie stipulates that the Free Public Libraries Act must be adopted, that a site must be given for the building, and that the cost must not be a burden on the penny rate.

### Public Notice.

### NOTICE.

*Mr. Robert Boyle, who was the proprietor of over four-fifths of the Shares in Robert Boyle & Son, Limited, Ventilating Engineers, 64 Holborn Viaduct, London, E.C., and 110 Bothwell Street, Glasgow, having acquired the remaining Shares, the business will in future be carried on by Mr. Boyle under its original title of*

**ROBERT BOYLE & SON.**

July 17th, 1903.

### A HANDBOOK FOR NURSES.

By Dr. WATSON.

Price **5/-** net, **5/4** post free.

This work is becoming more and more popular with the modern nurse, who finds that nowadays she is required to know much that is not included in the older text-books. Dr. Watson has succeeded in providing a complete guide to modern nursing.

### SURGICAL WARD-WORK AND NURSING.

By Dr. ALEXANDER MILES.

Price **3/6** net, **3/10** post free.

The Surgical Nurse will find this a most complete introduction to surgery and surgical instruments. It is profusely illustrated.

### THE HUMAN BODY.

By Professor BUEL COLTON.

Price **5/-** post free.

We cannot too highly recommend this most interesting work on the Physiology, Anatomy, and Hygiene of the Human Body. All three aspects are clearly and fully explained, and much information connected with the subject is also given. It is not merely a text-book; the intelligence and imagination of the student are constantly appealed to, and useful hints are also given to teachers.

### THE ART OF MASSAGE.

By A. CREIGHTON HALE.

Price **6/-** post free.

In this work Mrs. Creighton Hale has embodied her vast experience as a teacher, and has made her work complete by numerous and excellent illustrations.

### THE NURSES' DICTIONARY OF MEDICAL TERMS.

By HONNOR MORTEN.

Price **2/-** post free.

This is an invaluable companion for every nurse.

### OPHTHALMIC NURSING.

By SYDNEY STEPHENSON, F.R.C.S.E.

Price **3/6** net, **3/10** post free.

An admirable guide to the nursing of eye cases.

*These books are obtainable of all Booksellers, and are published by* THE SCIENTIFIC PRESS, LIMITED, 28 & 29 Southampton Street, Strand, London, *who will send their latest Catalogue of Nursing Text-books post free to any Nurse on application.*

